# Muscle-Derived Stem/Progenitor Cells Ameliorate Acute Kidney Injury in Rats through the Anti-Apoptotic Pathway and Demonstrate Comparable Effects to Bone Marrow Mesenchymal Stem Cells

**DOI:** 10.3390/medicina60010063

**Published:** 2023-12-28

**Authors:** Egle Pavyde, Arvydas Usas, Alius Pockevicius, Romaldas Maciulaitis

**Affiliations:** 1 Institute of Physiology and Pharmacology, Medical Academy, Lithuanian University of Health Sciences, LT-44307 Kaunas, Lithuania; egle.pavyde@gmail.com (E.P.); arvydas.usas@lsmuni.lt (A.U.); 2 Pathology Center, Department of Veterinary Pathobiology, Veterinary Academy, Lithuanian University of Health Sciences, LT-47181 Kaunas, Lithuania; alius.pockevicius@lsmu.lt; 3 Department of Nephrology, Medical Academy, Lithuanian University of Health Sciences, LT-50009 Kaunas, Lithuania

**Keywords:** muscle-derived stem progenitor cells, bone marrow mesenchymal stem cells, acute kidney injury, kidney regeneration

## Abstract

*Background and Objectives*: To date, the therapeutic potential of skeletal muscle-derived stem/progenitor cells (MDSPCs) for acute kidney injury (AKI) has only been evaluated by our research group. We aimed to compare MDSPCs with bone marrow mesenchymal stem cells (BM-MSCs) and evaluate their feasibility for the treatment of AKI. *Materials and Methods*: Rats were randomly assigned to four study groups: control, GM (gentamicin) group, GM+MDSPCs, and GM+BM-MSCs. AKI was induced by gentamicin (80 mg/kg/day; i.p.) for 7 consecutive days. MDSPCs and BM-MSCs were injected 24 h after the last gentamicin injection. Kidney parameters were determined on days 0, 8, 14, 21, and 35. *Results*: MDSPCs and BM-MSCs accelerated functional kidney recovery, as reflected by significantly lower serum creatinine levels and renal injury score, higher urinary creatinine and creatinine clearance levels (*p* < 0.05), lower TUNEL-positive cell number, and decreased KIM-1 and NGAL secretion in comparison to the non-treated AKI group. There was no significant difference in any parameters between the MDSPCs and BM-MSCs groups (*p* > 0.05). *Conclusions*: MDSPCs and BM-MSCs can migrate and incorporate into injured renal tissue, resulting in a beneficial impact on functional and morphological kidney recovery, which is likely mediated by the secretion of paracrine factors and an anti-apoptotic effect. MDSPCs were found to be non-inferior to BM-MSCs and therefore can be considered as a potential candidate strategy for the treatment of AKI.

## 1. Introduction

In recent years, a promising approach to managing acute kidney injury (AKI) has been the use of mesenchymal stem cells (MSCs), also known as mesenchymal stromal cells. MSCs are undifferentiated, self-renewable, non-hematopoietic adult stem cells of mesodermal origin [1]. These multipotent stem cells are broadly distributed in the body and can be relatively easily isolated from various tissues, such as bone marrow, umbilical cord blood, fat, muscle, periosteum, lung, testis, and dermis [2,3,4,5,6,7,8,9,10,11,12,13,14]. MSCs can differentiate into both mesenchymal and non-mesenchymal lineages, including differentiation toward adipocytes, chondrocytes, osteocytes, skeletal muscle cells, renal cells, and smooth muscle cells [9,10,11,15]. MSCs have been reported to promote regenerative kidney responses, leading to tissue repair and improved renal function in animal models of renal damage such as cisplatin, gentamicin, lipopolysaccharide, intramuscular glycerol, or ischemia-reperfusion-induced AKI [16,17,18,19]. These beneficial effects occur due to the anti-oxidative, anti-inflammatory, anti-apoptotic, and pro-angiogenic properties of the MSCs [17,18,20,21,22,23,24].

One of the most thoroughly investigated types of MSCs in renal regeneration is bone marrow mesenchymal stem cells (BM-MSCs) [25]. BM-MSCs have shown the ability to differentiate toward several renal cell lines, including mesangial cells and renal tubular epithelial-like cells [15,16]. BM-MSCs have also demonstrated their protective and regenerative capabilities by suppressing apoptosis, inflammation, and oxidative stress and increasing the production of growth factors such as VEGF, HGF, IGF-I, etc. [17,18]. Several pre-clinical studies have reported the efficacy of BM-MSCs in the treatment of AKI in different animal models. These studies have also investigated the possible mechanism of action of BM-MSCs in renal regeneration. While the Qian H et al. group has proposed that differentiation of BM-MSCs into renal tubular epithelial-like cells is a mechanism of action promoting renal repair, the majority of studies by other research groups support the indirect regenerative effect of BM-MSCs [16,17,18,22,23]. Such possible paracrine/endocrine effects of BM-MSCs result in functional and morphological renal recovery, defined by decreased kidney necrosis and fibrosis, decreased peak plasma creatinine and serum urea nitrogen, prolonged survival, and reduced mortality in the animals in experimental studies.

MSCs isolated from bone marrow are widely investigated in pre-clinical studies, and some early-phase clinical trials have also been completed [26,27,28,29]. However, a major limitation associated with the use of autologous BM-MSCs is their relatively long ex vivo cell expansion. Such a timeframe might range from 2 to 5 weeks, preventing the acute therapeutic use of autologous BM-MSCs. Furthermore, considering the future clinical application, even though BM-MSCs are accessible relatively easily and can be obtained from autologous sources, the source of stem cells is limited, and bone marrow biopsy is quite invasive and painful to the patient [2]. These hurdles encourage researchers to look for other sources of MSCs, such as adipose or skeletal muscle tissue.

MSCs derived from the postnatal skeletal muscle using the pre-plate isolation technique are known as skeletal muscle-derived stem/progenitor cells (MDSPCs) [3]. These slowly adhering cells supposedly reside in the perivascular niche [30]. MDSPCs are characterized by multipotency, long-term proliferation, and self-renewal capacities [31,32]. The most important properties of MDSPCs include their resistance to oxidative and inflammatory stress, induction of neovascularization, and stimulation of regeneration of various tissues, such as bone, cartilage, peripheral nerve, and skeletal muscles [33,34,35,36,37,38,39]. MDSPCs have also demonstrated the ability to extend life and health span in accelerated-aging animal models through paracrine/endocrine mechanisms of action [40]. Our research group has reported that MDSPCs contribute to kidney repair after AKI and can be considered a potential strategy for renal regeneration [41]. In this study, we aimed to compare the therapeutic effects of MDSPCs with BM-MSCs for the treatment of gentamicin-induced AKI.

## 2. Materials and Methods

### 2.1. Ethics

All experiments were performed using Wistar rats. The study-related procedures received approval from the Animal Health and Welfare Department, State Food and Veterinary Service of Lithuania (No. G2-21, 22 December 2014).

All animal experiments conducted comply with the ARRIVE guidelines and were carried out in accordance with EU Directive 2010/63/EU for animal experiments, the Animal Welfare and Protection Act of the Republic of Lithuania, Order no. B1-866, “Requirements for the keeping, care and use of animals used for scientific and educational purposes”, approved by the Director of the State Food and Veterinary Service on 31 October 2012, and Order no. B1-870, “On the establishment of the Lithuanian Experimental Animal Ethics Commission at the State Food and Veterinary Service and approval of its work regulations”, approved by the Director of the State Food and Veterinary Service on 6 November 2012.

### 2.2. Isolation and Characterization of MDSPCs and BM-MSCs

MDSPCs [3] and BM-MSCs [8] were isolated according to the previously published protocols. The characteristics of rat MDSPCs and BM-MSCs were assessed by population doubling time (PDT), flow cytometry, immunofluorescence, multipotent differentiation capacity, and RT-PCR, as previously described by our research group [41].

### 2.3. Nephrotoxicity Model

**Figure 1:**
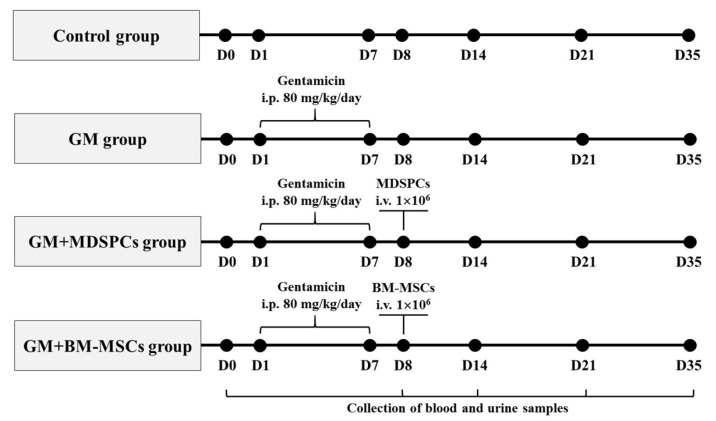
Acute kidney injury was induced through intraperitoneal (i.p.) administration of gentamicin (GM) at a dosage of 80 mg/kg daily for a continuous duration of 7 consecutive days. The rats were randomly allocated to one of four groups (n = 6 for each group at each time point): the control group (comprising healthy animals), the GM group (subjected to gentamicin injections alone), the GM+MDSPCs group (administered gentamicin injections followed by MDSPCs’ injection), and the GM+BM-MSCs group (administered gentamicin injections followed by BM-MSCs’ injection). A single injection of either MDSPCs or BM-MSCs (1 × 10^6^ cells/500 μL of serum-free medium) was intravenously (i.v.) administered via the tail vein 24 h after the final GM injection. Blood, urine, and tissue samples were collected at various time points, specifically on days 0, 8, 14, 21, and 35 of the experimental period, for the assessment of morphological and functional renal damage.

Experiments were conducted utilizing healthy female Wistar rats aged 8 to 12 weeks, with body weights ranging between 150 and 250 g. Animals were obtained from the Vivarium of the Veterinary Academy of the Lithuanian University of Health Sciences. Female rats were selected to demonstrate consistency with previous studies and to be able to make direct comparisons and decrease the heterogenicity of the response for this exploratory study [16,23,42,43]. Animals were housed in metabolic cages under standard conditions (temperature 24.0 ± 1 °C, humidity 50–55%) with a 12 h light/12 h dark cycle. Water and food were provided ad libitum. AKI was induced through daily intraperitoneal injections of gentamicin at a dosage of 80 mg/kg for a continuous period of 7 days. The AKI model design was based on our previous experiments [41,44]. The resource equation method was used to calculate a sample. Since there were 1 control and 3 study groups, the number of animals in one group had to be at least 5 [45]. To investigate the effects of MDSPCs and BM-MSCs in the gentamicin-induced AKI animal model, rats were randomly divided into 4 groups (n = 6 for each group per time point), as illustrated in Figure 1.

The control group animals were administered with daily i.p. injections of normal saline; the GM group was treated with gentamicin injections only, while the GM+MDSPCs and GM+BM-MSCs groups received gentamicin injections plus MDSPCs or BM-MSCs injections, respectively. A solitary injection of either MDSPCs or BM-MSCs (comprising 1 × 10^6^ cells suspended in 500 µL of serum-free medium) was intravenously administered through the tail vein, with this administration taking place 24 h subsequent to the final gentamicin injection. The stem cell concentration was chosen according to previously published protocols [46,47,48]. Hematological, urinary, and tissue specimens were obtained to assess renal function and tissue integrity. The rats were euthanized on day 8 for the verification of nephrotoxicity and AKI. Subsequently, euthanasia was performed on days 14, 21, and 35 of the experiment for further evaluation.

### 2.4. Biochemical Functional Analysis

Urine volume (V_U/24h_) was measured daily to track the onset and level of polyuria. Blood and 24 h urine samples were collected for the analysis of urinary (U_Cr_) and serum (S_Cr_) creatinine levels. Creatinine clearance (C_Cr_) was calculated as follows:

C_Cr_ = (U_Cr_ × V_U/24h_)/(S_Cr_ × 24 × 60)

Urine and blood samples were subjected to analysis utilizing the COBAS INTEGRA 400 plus automatic biochemistry analyzer (Tegimenta Ltd., Roche, Basel, Switzerland). Sample collection occurred at specific time points, namely, on day 0 (24 h preceding the initiation of gentamicin injection), day 8 (24 h following the final gentamicin injection), and subsequently on days 14, 21, and 35.

### 2.5. Biochemical Injury Markers KIM-1 and NGAL Secretion Analysis

For the detection of AKI urinary biomarkers, kidney injury molecule-1 (KIM-1) and neutrophil gelatinase-associated lipocalin (NGAL) were selected based on previously published data [49,50]. Changes in KIM-1 and NGAL in urine were determined using an enzyme-linked immunosorbent assay (ELISA). Six time points (days 0, 8, 11, 14, 21, and 35) were chosen for the determination of the serial changes in urinary protein secretion. ELISA kits were purchased from Abcam, and the assay was performed according to manufacturer instructions. Plates were scanned using a Tecan microplate reader (Tecan Trading AG, Männedorf, Switzerland) with an excitation wavelength of 450 nm and analyzed with Magellan Data Analysis Software version 6.

### 2.6. Renal Histology

Kidney specimens were taken on days 0, 8, 14, 21, and 35. Fixation was performed using 10% buffered formalin in a ratio of 1:20 and within a 24–48 h period embedded in paraffin. The tissue specimens were sectioned to a thickness of 5 µm and subsequently subjected to hematoxylin and eosin (HE) staining for the purpose of light-microscopy analysis. The histological assessment was carried out independently by three researchers who were blinded to the experimental conditions. The evaluation of tubular injury encompassed the assessment of specific histopathological features, including tubular necrosis, loss of brush border, cast formation, and tubular dilatation. These evaluations were conducted in ten randomly selected, non-overlapping fields within each tissue section. The scoring system employed for these assessments was as follows: 0 (0% affected), 1 (≤10% affected), 2 (11–25% affected), 3 (26–45% affected), 4 (46–75% affected), and 5 (76–100% affected). To calculate the tubular injury score, the percentage of affected tubules within each field was determined, considering the total number of tubules present as the standard reference. The percentage of tubular injuries was calculated for each field using the following formula:

Renal injury (%) = (Number of injured tubules/Number of whole tubules) × 100

### 2.7. Apoptosis Assay

Apoptosis was determined using a terminal deoxynucleotidyl transferase dUTP nick end-labeling (TUNEL) assay kit (Molecular Probes Inc., USA) following the instructions provided by the manufacturer on frozen kidney sections. TUNEL-positive, apoptotic tubular epithelial cells were counted in ten non-overlapping fields per section in the renal cortex (x400). Apoptotic cell presence was quantified through the calculation of the percentage of cells exhibiting TUNEL positivity. The total cell count per field was employed as the baseline standard for this assessment. The determination of the percentage of TUNEL-positive cells in each field was executed as follows:

Apoptosis score (%) = (Number of TUNEL-positive cells/Number of all cells) × 100

### 2.8. In Vivo Tracking of MDSPCs and BM-MSCs

For the purpose of in vivo tracking, both MDSPCs and BM-MSCs were labeled with the red fluorescent membrane dye PKH26, following the manufacturer’s instructions provided by Sigma-Aldrich (Burlington, MA, USA), immediately before their injection. Subsequently, the rats were euthanized at specific time points, specifically at 7, 14, and 28 days following MDSPCs’ administration, corresponding to days 14, 21, and 35 of the experiment. Kidney samples were snap frozen and preserved at −80 °C until further analysis. To facilitate examination, the frozen tissue samples were sectioned into 5 µm slices, and the nuclei were counterstained with 4′,6-diamidino-2-phenylindole (DAPI). Fluorescent microscopy was conducted utilizing an Olympus lX73 microscope, coupled with the use of QCapture Pro version 7 image and analysis software.

### 2.9. Statistical Analysis

Statistical data analysis was conducted employing SPSS Statistics version 17.0 software. Quantitative data were presented as the mean ± standard deviation (SD). A Student’s *t*-test was employed to compare data between two groups, while an analysis of variance (ANOVA) followed by the Bonferroni post hoc test was utilized for multigroup comparisons. A significance threshold of *p* < 0.05 was established to denote statistical significance.

## 3. Results

### 3.1. Characteristics of MDSPCs and BM-MSCs

Morphologically, MDSPCs exhibited diverse shapes, appearing as small, round, triangular, or spindle-shaped cells. In contrast, BM-MSCs, during the initial day of culture, manifested a round morphology, which transitioned to a spindle-shaped appearance after 3–4 days. MDSPCs demonstrated a significantly lower population doubling time (43.64 ± 3.10 h) when compared to BM-MSCs (60.78 ± 3.34 h), a distinction that was statistically significant (*p* = 0.001). Furthermore, both MDSPCs and BM-MSCs, which were derived from rat sources, exhibited notable positivity for CD90 and CD59 markers, while displaying a lack of expression for CD34 and CD45 markers. Additionally, MDSPCs exhibited positivity for Desmin, indicating their origin from muscle tissue, and a weakly positive expression of c-kit (CD117), whereas BM-MSCs did not express these markers. MDSPCs displayed a significantly higher expression of OCT4 in comparison to BM-MSCs, while the expression levels of NANOG and SOX2 were found to be similar in both cell lineages. Importantly, both cell types exhibited the capacity for adipogenic, chondrogenic, osteogenic, and myogenic differentiation in vitro. For a more comprehensive account of the detailed characteristics of MDSPCs and BM-MSCs, readers are referred to our prior scientific publication [41].

### 3.2. Effects of MDSPCs and BM-MSCs on Kidney Function after AKI

We selected five specific time points (days 0, 8, 14, 21, and 35) to monitor the sequential alterations in serum and urinary creatinine levels as well as creatinine clearance in rats. In the control group of animals, there were no statistically significant differences observed in any of these parameters at any of the time points. Similarly, on day 0, there were no discernible statistically significant distinctions detected among the four groups. Moreover, on days 0, 8, and 14, no statistically significant differences were observed in any of the functional parameters between the GM, GM+MDSPCs, and GM+BM-MSCs groups (*p* > 0.05).

Physiological and functional alterations were evident in the GM, GM+MDSPCs, and GM+BM-MSCs groups on days 8 and 14, indicative of acute kidney injury induced by gentamicin. These variations were statistically significant when compared to the parameters recorded on day 0 within each respective group (*p* < 0.05), as well as in comparison to the control group on days 8 and 14 (*p* < 0.05).

**Figure 2:**
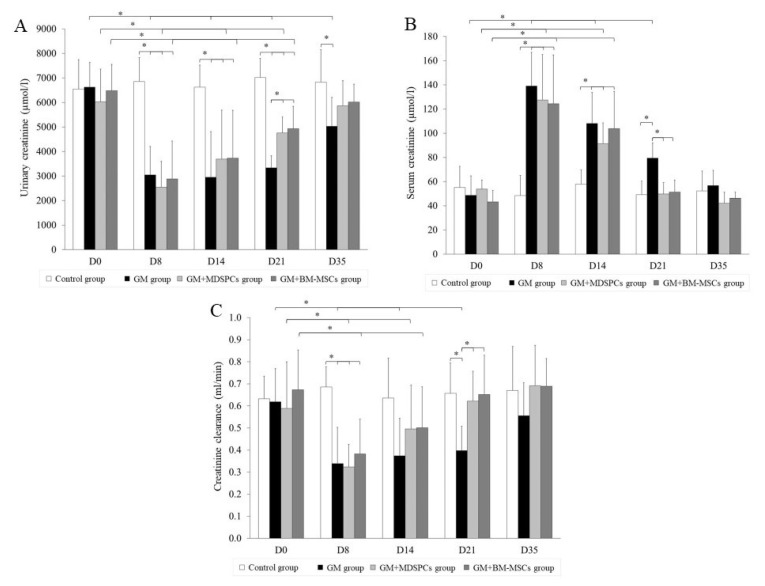
MDSPCs’ and BM-MSCs’ impact on kidney function after acute kidney injury. Gentamicin-induced acute kidney injury was confirmed by serum and urinary creatinine levels and creatinine clearance in GM, GM+MDSPCs, and GM+BM-MSCs group animals ((**A**–**C**) D8 and D14). Both MDSPCs and BM-MSCs demonstrated the ability to expedite the recovery of renal function following kidney injury. This was evident through several key indicators, including a notably elevated urinary creatinine level (**A**), enhanced creatinine clearance (**C**), and a significant reduction in serum creatinine levels (**B**) observed on day 21 when compared to rats in the GM group, which did not receive MDSPCs treatment (*p* < 0.05). * *p* < 0.05, significant difference.

As depicted in Figure 2, the administration of MDSPCs and BM-MSCs (in the GM+MDSPCs and GM+BM-MSCs groups, respectively) exerted a notable impact on the recovery of kidney function following acute kidney injury (AKI). This was evidenced by a significant decrease in serum creatinine levels (*p* < 0.05), an increase in urinary creatinine levels (*p* < 0.05), and an improvement in creatinine clearance (*p* < 0.05), as compared to the GM group rats that did not receive MDSPCs or BM-MSCs treatment.

### 3.3. Effects of MDSPCs and BM-MSCs on KIM-1 and NGAL Secretion

**Figure 3:**
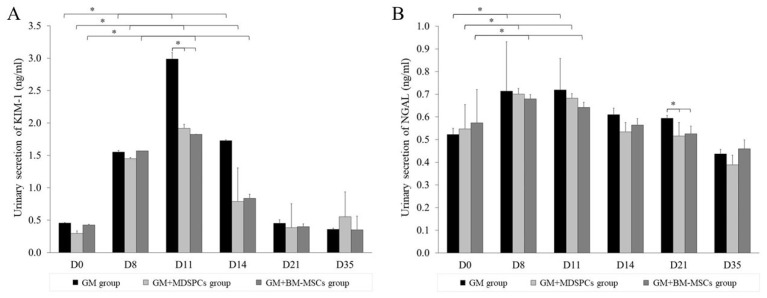
MDSPCs’ and BM-MSCs’ impact on the urinary secretion of kidney biomarkers KIM-1 and NGAL. Gentamicin-induced acute kidney injury was confirmed by urinary secretion of kidney biomarkers KIM-1 and NGAL in GM, GM+MDSPCs, and GM+BM-MSCs group animals ((**A**,**B**) day 8). Both MDSPCs and BM-MSCs accelerated functional kidney recovery after renal damage, as reflected by the significantly lower KIM-1 secretion on day 14 (*p* < 0.05; A; D14) and NGAL secretion on day 21 (*p* < 0.05; B; D21), compared with the GM group rats. * *p* < 0.05, a significant difference.

The quantification of urinary KIM-1 and NGAL via ELISA revealed a statistically significant elevation in the levels of these proteins in the urine of rats treated with 80 mg/kg of gentamicin. The increase in urinary proteins was statistically significant after 7 days of consecutive gentamicin injections in GM, GM+MDSPCs, and GM+BM-MSCs group animals, demonstrating gentamicin-induced acute kidney injury. As shown in Figure 3, both MDSPCs and BM-MSCs had a statistically significant beneficial effect on kidney function, as reflected by lower KIM-1 secretion on day 11 (*p* < 0.05) and numerically on day 14. The trends in lower NGAL secretion were observed starting from day 11 and statistically significant on day 21 (*p* < 0.05), compared with the GM group rats treated with neither MDSPCs nor BM-MSCs. The beneficial effect was more expressed by measuring KIM-1 protein as compared to NGAL.

### 3.4. Effects of MDSPCs and BM-MSCs on Kidney Histology after AKI

**Figure 4:**
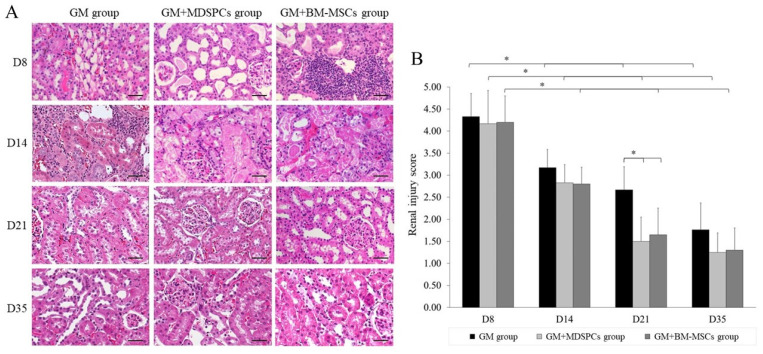
MDSPCs’ and BM-MSCs’ impact on morphological recovery after acute kidney injury. On day 8, evidence of acute kidney injury was evident across all three study groups that received gentamicin treatment, as indicated by the presence of tubular necrosis, cast formation, loss of brush border in the renal tubules, and tubular dilatation. Treatment with stem cells resulted in a significant reduction in renal tubular damage, as evidenced by the histological examination of kidney tissue (**A**), along with a notably lower renal injury score (**B**) in the GM+MDSPCs and GM+BM-MSCs groups (*p* < 0.05). This reduction was in comparison to the injury score observed in the GM group on day 21 of the experiment, which was two weeks after the injection of MDSPCs and BM-MSCs, respectively. Images are 20× magnification (scale bar is 50 μm). * *p* < 0.05, a significant difference.

Administration of gentamicin at a dosage of 80 mg/kg for a continuous duration of 7 consecutive days induced characteristic features of aminoglycoside-induced AKI, including tubular necrosis, cast formation, loss of brush border in the renal tubules, and tubular dilatation. Although a reduction in kidney injury was observed on day 14 (7 days after the final gentamicin injection) in the GM+MDSPCs and GM+BM-MSCs groups compared to the GM group, this difference did not reach statistical significance (*p* > 0.05). The treatment with stem cells notably ameliorated renal tubular damage, as evidenced by the kidney histology (Figure 4A) and a significantly reduced renal injury score (Figure 4B) in the GM+MDSPCs and GM+BM-MSCs groups (*p* < 0.05), when compared to the injury score observed in the GM group on day 21 of the experiment, which was two weeks after the administration of MDSPCs and BM-MSCs, respectively.

### 3.5. Anti-Apoptotic Effects of MDSPCs and BM-MSCs

**Figure 5:**
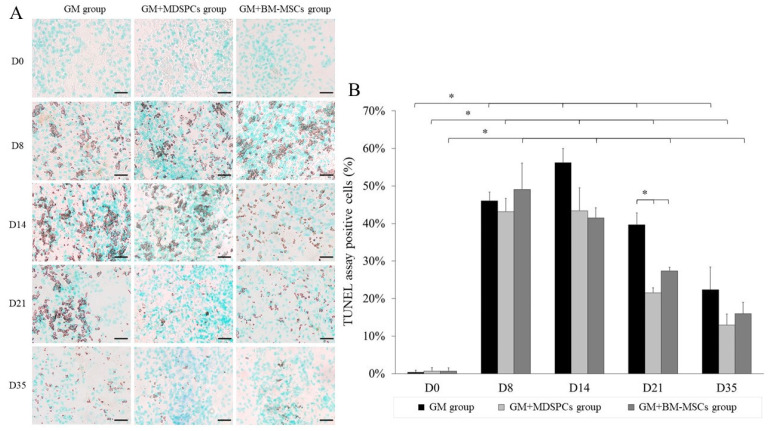
Anti-apoptotic effect of MDSPCs and BM-MSCs treatment after acute kidney injury. Anti-apoptotic effect evaluated using TUNEL assay showed a significantly reduced percentage of apoptotic cells (visible as brown to black colored in kidney histology (**A**) and significantly more proportionally seen (**B**) in GM+MDSPCs (*p* < 0.05) and GM+BM-MSCs groups (*p* < 0.05) as compared to GM group on day 21 and day 35. Images are 10X magnification (scale bar is 100 μm). * *p* < 0.05, a significant difference.

The results indicate that both mesenchymal stem cell types, MDSPCs and BM-MSCs, promote regeneration and exert an anti-apoptotic effect on gentamicin-induced nephrotoxicity (Figure 5).

Although the numerically TUNEL assay showed reduced apoptosis already at day 14 (i.e., after one week after the injection of MDSPCs and BM-MSCs), statistically significantly reduced percentages of apoptotic cells were observed on day 21 in MDSPCs- (*p* < 0.05) and BM-MSCs (*p* < 0.05)-treated animal kidney sections as compared to the GM group (GM+MDSPCs 21.53 ± 1.34%, GM+BM-MSCs 27.35 ± 0.97% vs. GM group 39.66 ± 3.14% TUNEL-positive cells) and on day 35 (GM+MDSPCs 12.99 ± 2.90%, GM+BM-MSCs 16.02 ± 2.95% vs. GM group 22.38 ± 5.98% TUNEL-positive cells).

### 3.6. Tracking of MDSPCs and BM-MSCs In Vivo

**Figure 6:**
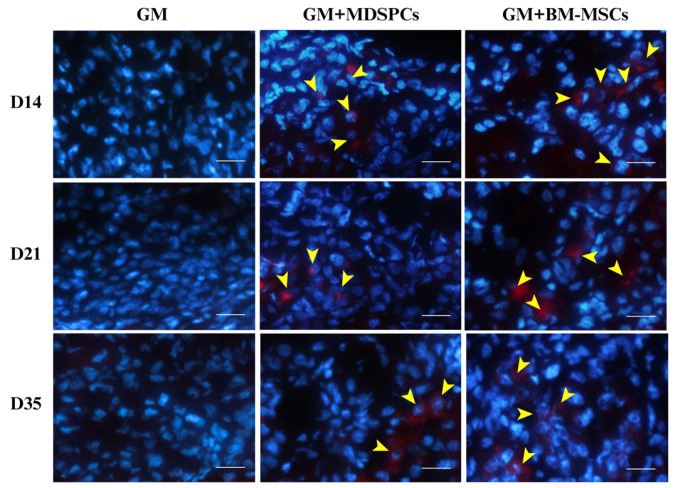
Tracking of rat muscle-derived stem/progenitor cells (MDSPCs) and bone marrow mesenchymal stem cells (BM-MSCs) through the injured kidneys induced with gentamicin. Our observations revealed the presence of PKH-26-labeled MDSPCs and BM-MSCs within the renal cortex on days 14, 21, and 35 post-treatment (marked with yellow arrows). These cells were identified within the renal cortex and were primarily localized in proximity to renal tubules and within the interstitial compartment of the kidney. The red staining represents PKH-26-labeled stem cells, while the blue staining represents the cell nuclei, which were stained with DAPI. Images are 20× magnification (scale bar is 50 μm).

We assessed the presence of MDSPCs and BM-MSCs within renal tissue by examining the kidney sections for PKH-26-labeled cells on days 14, 21, and 35 of the experimental timeline. PKH-26-labeled MDSPCs and BM-MSCs were discernible within the renal cortex, where they were primarily localized around renal tubules and within the interstitial compartment of the kidney, as illustrated in Figure 6. In contrast, no PKH-26-positive cells were identified in the renal tissue of the GM group on days 14, 21, and 35. Moreover, we did not observe any discernible trends indicating a decline in the number of engrafted cells during the observed time frame, whether it pertained to MDSPCs or BM-MSCs.

## 4. Discussion

In the era of stem cell research, mesenchymal stem cells have been one of the key sources of cell-based therapies [51]. Different research groups identified, isolated, and compared the cellular morphology, surface markers, and differentiation abilities of MSCs derived from different sources [2,3,4,5,6,7,8,9,10,11,12,13,14,15]. A lot of work has been performed to date to investigate the possible therapeutic effects of MSCs in different acute kidney injury models [16,17,18,19,20,21,22,23,24]. In this study, we directly compared the therapeutic potential of MSCs derived from skeletal muscle and bone marrow in the setting of gentamicin-induced acute kidney injury. Before that, we reported a full characterization of these different types of MSCs [41].

The present study shows that the administration of MDSPCs in a rat model of gentamicin-induced AKI resulted in rapid improvement of renal function and attenuation of histologically proven renal damage. We speculate that these therapeutic effects of MDSPCs might be predominantly mediated through their ability to migrate to the damaged kidney tissue, incorporate into the renal cortex, and exert an anti-apoptotic effect. Moreover, the therapeutic effect of MDSPCs was found to be at least non-inferior to the effect of BM-MSCs. To our knowledge, this represents the first study in the literature on the comparison of the therapeutic effects of MDSPCs and BM-MSCs in an animal model of gentamicin-induced AKI.

A major issue that remains undiscovered is the determination of the most effective stem cell source in different disease models. In the present study, we have compared the therapeutic effects of MDSPCs and BM-MSCs on renal recovery after AKI. Both MDSPCs and BM-MSCs exhibited the capacity to mitigate kidney dysfunction and alleviate renal injury induced by gentamicin. This was evident through several key indicators, including a significant reduction in serum creatinine levels, an increase in urinary creatinine levels, and an enhancement in creatinine clearance when compared to the animals in the GM group. Moreover, both cell therapies have shown statistically significant results in the reduction in the renal biomarkers KIM-1 and NGAL urinary secretions, as well as the renal injury score, in comparison to the non-treated rats. No superiority could be found in this comparative study of AKI treatment with MSCs from different sources regarding biochemical and morphological renal changes, proposing that both therapies should be considered comparably effective. However, looking at the clinical perspectives of both therapies, the superiority may fall to the treatment of MDSPCs due to several reasons. As previously documented, MDSPCs possess a shorter population doubling time, which means that the necessary quantity of cells for therapeutic applications can be generated within a more expedited timeframe [41,52]. Considering the clinical application of these cellular therapies using autologous stem cells, skeletal muscle tissue biopsy would be less invasive and less painful in comparison to bone marrow biopsy, therefore better tolerated by the patients.

To further understand the cytokinetic properties of MDSPCs and the mechanisms underlying the effects of this stem cell lineage in damaged renal tissue, MDSPCs were tracked in vivo. Stem cells were labeled with the PKH-26 red fluorescent cell membrane labeling dye. The results revealed that only a small number of positive cells were found to be located around the kidney tubules in each section (Figure 5). However, PKH-26-labeled MDSPCs were still present in the renal cortex 4 weeks after injection. Although previous studies indicated that mesenchymal stem cells could differentiate into renal epithelial or mesangial cells [15,16], recent evidence suggests that their direct incorporation into kidney tissue and differentiation toward renal lineages might not play a key role in kidney recovery after AKI. The therapeutic effect of MSCs is most probably manifested through the paracrine/endocrine pathway, resulting in the reduction of apoptosis, inflammation, and oxidative stress [17,18,20,21,22,23,24]. MDSPCs, in particular, have been previously shown to reduce fibrosis, increase angiogenesis, and reduce inflammation [34,53,54]. Trophic factors that might be key in the kidney recovery process include vascular endothelial growth factor (VEGF), fibroblast growth factor 2 (FGF2), insulin-like growth factor-1 (IGF-1), or hepatocyte growth factor (HGF) involved in the inhibition of the toll-like receptors (TLR4)-nuclear factor-kappa B (NF-κB) signaling pathway, and tumor necrosis factor-inducible gene 6 protein (TSG-6), due to its effect on promoting renal tubular epithelial cell proliferation by modulating inflammation [17,18,53,54]. Trophic factors involved in this recovery process shall be further investigated.

Tubular cell apoptosis is a characteristic feature of gentamicin-induced AKI, which results in kidney dysfunction. As mentioned earlier, the anti-apoptotic effect of MSCs plays a crucial role in kidney recovery after AKI [20,21]. In the present study, the number of TUNEL-positive (apoptotic) cells increased significantly after the gentamicin injections for 7 consecutive days. Both cell therapies (with MDSPCs and BM-MSCs) resulted in a significant reduction in TUNEL-positive cells in the renal tissue on day 21 (2 weeks after stem cell injection). The apoptotic processes were evident in much larger areas in the GM group as compared to the GM+MDSPCs and GM+BM-MSCs groups until the end of the experiment. These findings suggest that the regenerative effects of both MDSPCs and BM-MSCs may rely on their anti-apoptotic capacity. Such an anti-apoptotic mechanism of action would also explain the beneficial role of MSC therapy in kidney regeneration, even though only a small number of cells were found present in the renal cortex.

We have demonstrated at least the non-inferiority of MDSPCs’ application for the treatment of AKI in comparison to BM-MSCs’. Apart from these novel findings, several questions were not addressed in this study and need further investigation. First of all, our research group has not investigated different doses of MSCs or multiple administrations at different time points [24,55]. These issues were not essential to be resolved in order to reach our main objective—to demonstrate the principal comparability of MDSPCs with historical comparator BM-MSCs. For the AKI treatment after maximal insult, however, it would be important to investigate prior to moving into the clinic. The dose dependence could strengthen the signal of effect, although the translation to clinical application could start with the scaling of our selected dosage for the treatment as well as defining the best time for the administration. Secondly, further research shall address the questions related to the safety issues for application in humans, such as infections (microbial contamination of starting materials), tumorigenicity, immunogenicity (rejection), and ectopic tissue formation [56]. Thirdly, while we have evaluated the anti-apoptotic effect of MSCs derived from bone marrow and skeletal muscle, we have not investigated other possible mechanisms of action, starting with defining particular anti-inflammatory and anti-oxidative effects. We do not expect that these effects would be essentially different from the findings of other research groups [18,20,21,24,46]. Also, an increasing number of recent studies have demonstrated that MSC-derived exosomes have innate therapeutic potential, which was not investigated in our research [57,58,59]. Longer follow-up periods and evaluations of life span could also provide valuable information on the long-term outcomes of MSC therapy.

## 5. Conclusions

The current study shows that both MDSPCs and BM-MSCs have the ability to migrate and incorporate into injured renal tissue, resulting in a beneficial impact on functional and morphological kidney recovery, which is likely to be mediated by the secretion of trophic factors and their anti-apoptotic effect. The utilization of MDSPCs in cellular therapy holds promise as a prospective strategy for the experimental treatment of acute kidney injury (AKI) and merits further investigation. The following research shall be focused on investigating different doses of MDSPCs, multiple administrations at different time points, anti-inflammatory and anti-oxidative elements in their mechanisms of action, as well as the effects of MDSPCs-derived exosomes. Altogether, this work could not only provide new scientific evidence for revealing the potential of MDSPCs in kidney regeneration but also eventually benefit MDSPCs-based clinical therapy for acute kidney injury patients.

## Data Availability

The data used to support the findings of this study are available from the corresponding author upon reasonable request.
